# Combatting Social Isolation Among Older Adults in a Time of Physical Distancing: The COVID-19 Social Connectivity Paradox

**DOI:** 10.3389/fpubh.2020.00403

**Published:** 2020-07-21

**Authors:** Matthew Lee Smith, Lesley E. Steinman, E. A. Casey

**Affiliations:** ^1^Center for Population Health and Aging, Texas A&M University, College Station, TX, United States; ^2^Department of Environmental and Occupational Health, School of Public Health, Texas A&M University, College Station, TX, United States; ^3^Health Promotion Research Center, School of Public Health, University of Washington, Seattle, WA, United States; ^4^Evidence-Based Leadership Collaborative, United States; ^5^AARP Foundation, Washington, DC, United States

**Keywords:** social isolation, loneliness, social connectivity, paradox, distanced connectivity, screening, service provision, aging network

## Abstract

Social isolation is an important public health issue that has gained recognition during the COVID-19 pandemic because of the risks posed to older adults based on physical distancing. The primary purposes of this article are to provide an overview of the complex interconnectedness between social isolation, loneliness, and depression while introducing the COVID-19 Connectivity Paradox, a new concept used to describe the conflicting risk/harm continuum resulting from recommended physical distancing. In this context, examples will be provided for practical and feasible community-based models to improve social connectivity during COVID-19 by adjusting the processes and modalities used to deliver programs and services to older adults through the aging social services network. The COVID-19 pandemic has highlighted the need for clinical and community-based organizations to unite and form inter-sectorial partnerships to maintain the provision of services and programs for engaging and supporting older adults during this difficult time of physical distancing and shelter-in-place and stay-at-home orders. The aging social services network provides a vital infrastructure for reaching older underserved and/or marginalized persons across the U.S. to reduce social isolation. Capitalizing on existing practices in the field, older adults can achieve distanced connectivity to mitigate social isolation risk while remaining at safe physical distances from others.

## Introduction

Prior to COVID-19, social isolation among older adults was a major public health issue gaining international recognition as being detrimental to quality of life and premature mortality. As social beings, our social relationships (both quality and quantity) largely impact our health and well-being, as well as risk for illness and death (1). While social support has a long-standing determination as a key social determinant of health (SDOH) (2), social isolation, whether perceived or actual, has only recently emerged as a recognized SDOH. The negative ramifications of social isolation and low social connectivity have been equated to the health risks of high blood pressure, physical inactivity, obesity, or smoking 15 cigarettes a day (3–5).

Older adults are particularly vulnerable to social isolation because of aging-related role transitions (e.g., retirement, caregiving, loss of family/friends), physical changes (e.g., changes in health status, mobility, sensory function), and societal views (e.g., ageism). Despite the progress made combatting social isolation and loneliness by organizations across the healthcare sector, aging services network, and public health system, the fight against social isolation remains in its infancy. And, in the new era of the COVID-19 pandemic, innovative, and effective efforts to blunt the impacts of social isolation and bolster social connectivity are more critical than ever before.

At the time of this writing, the United States has the largest burden of COVID-19 confirmed cases and deaths worldwide (6, 7). Older adults are especially at risk for COVID-19 complications because they have higher rates of disease and co-morbidities, on average, compared to younger adults (8, 9). Chronic disease, coupled with the biological and physiological changes associated with aging, make older adults particularly susceptible to COVID-19 transmission, severe illness response, and diminished recovery.

To remain safe from the virus, older adults must strictly limit their contact with others through physical distancing (i.e., remaining 6 or more feet from others, also known as social distancing) (10). This plus shelter-in-place and stay-at-home orders limit interactions with family, friends, caregivers, and organizations. While obviously helpful to prevent exposure to and the spread of COVID-19, limited physical interactions with others directly softens (or negates) ongoing efforts to reduce social isolation and improve connectivity among older adults. Herein lies the basis of the COVID-19 Social Connectivity Paradox. How do we quickly and effectively modify our existing strategies to improve connectivity in a time of recommended and required physical distancing? How do we take the “human” out of human services in clinical and community settings? How do we introduce and implement opportunities for meaningful connectivity without physical interactions? How do we capitalize on the strengths of older adults and their contributions to society during crisis to ensure they support their loved ones and facilitate connectivity among their peers?

Although in no way intended to be a comprehensive review, the purposes of this article are to: (1) provide a definition and overview of the complexities of social isolation and its interconnectedness with loneliness and depression; (2) explain the COVID-19 Connectivity Paradox, a new concept used to describe the conflicting risk/harm continuum resulting from recommended physical distancing; (3) highlight screeners and assessments needed to rapidly and accurately identify older adults at-risk for social isolation; and (4) provide examples of practical and feasible community-based models to improve social connectivity during COVID-19 by adjusting the processes and modalities used to deliver programs and services to older adults through the aging social services network. Older persons who are vulnerable to COVID-19 are also vulnerable to social isolation. Therefore, this article aims to offer practical solutions for use in the aging social services network so older marginalized persons can avoid further health problems and inequities resulting from the COVID-19 pandemic.

## Definitions

Social isolation can be defined as the “relative absence of social relationships” (11). It is an objective measure that describes a physical separation from people and can be quantified by looking at the size of one's social network, level of social integration (e.g., belonging to social groups or a faith community; frequency of social contacts), and whether one lives alone or is partnered (12, 13). Late-life social isolation has been linked to poor health, depression, cognitive decline, and mortality (3), and the lack of social contact among older adults was recently associated with $6.7 billion in additional Medicare spending annually (14, 15).

Loneliness can be defined as perceived isolation (16), or a disconnect between social ties an older adults has and those they want (17). This feeling of being alone can be accompanied by distress that results from the discrepancies between ideal and perceived social relationships (18). However, it is important to note that being alone does not always yield negative feelings (i.e., one can be alone but not lonely) (19). Loneliness can be emotional (e.g., negative feelings because of not having a companion or emotional support) or social (e.g., negative feelings because of a perceived lack of a wider social network) (16). Like with social isolation, older persons who are lonely have greater risk of negative functional and health outcomes and premature death (20).

Depression in later life is well-documented and can be assessed by well-validated tools. Depression is a substantial public health issue. The World Health Organization identified depression as the leading cause of disability worldwide, citing a 20% increase over the last decade (21). Approximately 15–27% of older adults experience depressive symptoms (22), and the burden is higher for more marginalized older adults who receive social services (23). Late-life depression has been associated with reduced quality of life and function, poor self-rated health, excess service utilization, and increased disability, morbidity, and mortality, including suicide (24–27).

The above conditions of social isolation, loneliness, and depression represent overlapping yet distinct expressions and experiences among older adults. These conditions are interrelated and interconnected, can manifest sequentially or in concert, and have the ability to intensify one another. For example, a known risk factor for late-life depression is the increasing isolation due to role changes as one ages (e.g., retirement, caregiving, widowhood, declining mobility) (28–31). Additionally, the co-occurrence of social isolation and loneliness is largely documented, and while commonly not disentangled, collectively have ramifications for behavioral and mental health as well as all-cause mortality (32–34). Therefore, situations and events that cause one of these conditions can also evoke the other conditions simultaneously or sequentially. As such, efforts to combat any one of these conditions may also have larger impacts on the entirety of these conditions, dependent upon their existence, degree of severity, and the intervention/strategy/solution employed.

Furthermore, while social isolation and loneliness are often used interchangeably, each describes different aspects of lacking or limited social connectedness. Therefore, it may be more appropriate to focus on older adults' lack of social connectedness to more accurately pinpoint the root issues faced by the older adult and more appropriately introduce interventions and solutions to mitigate the problem. Social connectedness represents the structural (e.g., network size, marital status), functional (e.g., perceived social support, loneliness), and quality (e.g., positive or negative such as relationship quality or strain) aspects of social relationships (35, 36).

Several identified factors and mechanisms indicate that a lack of social connectedness can impact health (13). Quantifiable or qualitative lack of social connections can impact an older adult's lifestyle (e.g., physical activity, nutrition, sleep, smoking, risk-taking behavior like substance use) or their adherence and compliance with managing health (e.g., taking medications, following recommended changes to diet, physical activity, and substance use). To design and deliver appropriate and effective public health interventions to improve connectivity, efforts are needed to clearly identify and specify the type of social disconnection and the pathway by which it impacts health. This is especially important in the time of COVID-19 in that the structural, functional, and quality aspects of social connectedness have been disrupted by shelter-in-place and stay-at-home orders as well as fear of infection and conscious efforts to remain physically distanced.

## The COVID-19 Social Connectivity Paradox

**Figure 1:**
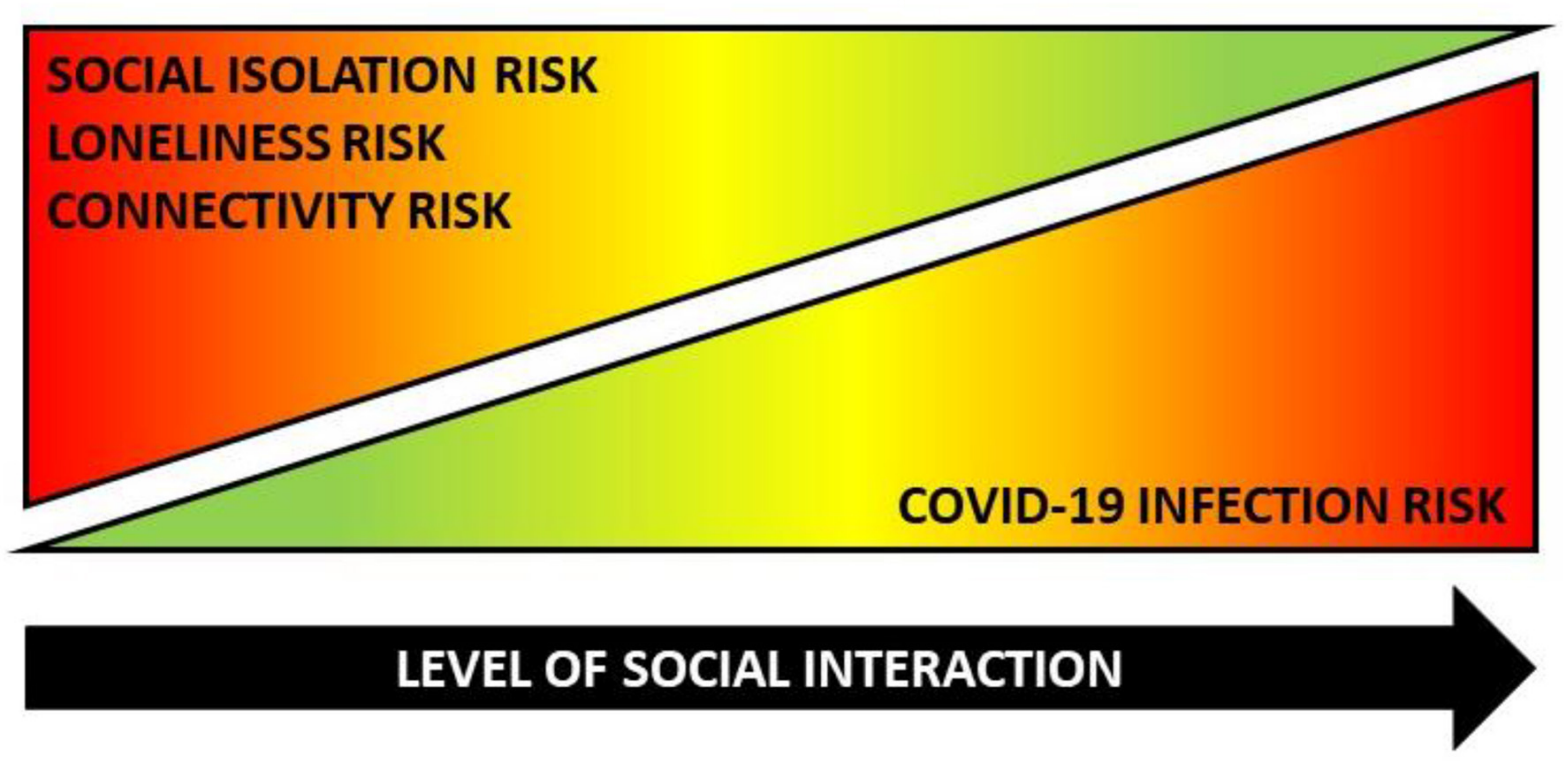
The COVID-19 social connectivity paradox.

As described above, meaningful interactions with others as well as objective and subjective elements of connectedness are important to the physical and mental health of older adults. Many older adults stay quite active: they continue to work, take care of grandchildren, volunteer in community organizations, provide caregiver support to spouses or other relatives, and engage with friends and family. However, COVID-19-related physical distancing recommendations and orders to shelter-in-place and stay-at-home have directly interrupted older adults' social connectivity in terms of structure, function, and quality. To protect themselves, older adults must avoid the people, places, and services they rely on for companionship, support, and resources. Based on their higher COVID-19-related vulnerability, many older adults have limited physical and social interactions with loved-ones and the people they rely on for support. Many older adults have also restricted their patronage to businesses, community organizations, and healthcare facilities for safety reasons (whether by their own decision or because of temporary establishment closures). While it is encouraging that older adults have followed recommendations to limit human contact to avoid COVID-19 exposure, these altered and truncated interaction patterns greatly diminish social connectedness and increase older adults' risk for social isolation, loneliness, and depression. As such, the COVID-19 Social Connectivity Paradox posits that a common set of actions simultaneously protects and harms older adults during this pandemic. More specifically, the paradox postulates that as the level of an older adult's physical interactions with others increases, it can protect against social isolation and disconnectedness, although it can increase the risk of COVID-19 exposure. Conversely, as the level of an older adult's physical interactions with others decreases, it can increase risk for social isolation and disconnectedness, although it can protect against risk of COVID-19 exposure. As depicted in Figure 1, within the COVID-19 Social Connectivity Paradox, a common action (interacting with others) can simultaneously increase risk (illustrated in red) for one risk factor while diminishing risk (illustrated in green) for another.

While the COVID-19 Social Connectivity Paradox itself is logical, its ramifications warrant attention because it is intensifying the effects and magnitude of social isolation, disconnectedness, and associated mental health issues. Based on news and social media reports, older adults are keenly aware that they are at higher risk for severe morbidity and mortality from COVID-19. Such heightened awareness causes older adults to be more diligent and vigilant about protective measures against the virus, but it also limits their social mobility and connectivity while evoking fear and anxiety. Therefore, to avoid COVID-19 exposure, older adults must knowingly or unknowingly place themselves at risk for social isolation and disconnectedness. While physical distancing during COVID-19 may initiate social isolation risk among many older adults based on the abrupt and severe nature of the situation, the ramifications of this risk may be dramatically accelerated and/or exacerbated for older adults who were already experiencing social isolation and limited connectedness before the COVID-19 pandemic. The ramifications of COVID-19 Social Connectivity Paradox will be seen for months or years to come, based on the estimated duration of COVID-19-related physical distancing and the projected resurgence of COVID-19 during cold and flu seasons. As such, immediate solutions are needed to improve social connectivity and connectedness among older adults both now and beyond the time of pandemic cautions.

## Progression Toward Leveraged, Action-Oriented Risk Screening and Assessment

A variety of validated scales and measures exist to assess social isolation and associated concepts (e.g., loneliness, social integration, disconnectedness). Each tool was developed to examine a specific concept or construct within these interconnected and overlapping concepts. Each contains its own set of items and is used to identify the presence of the concept or construct and its associated risk. Examples of existing of commonly used, validated assessment tools include the Berkman-Syme Social Network Index (37), Revised UCLA Loneliness Scale (38, 39), Duke Social Support Index (40), Lubben Social Network Scale (41), de Jong Gierveld Loneliness Scale (42, 43), Cornwell Perceived Isolation Scale (44), and Campaign to End Loneliness Measurement Tool (45). Such tools are extremely valuable at identifying risk when appropriately and purposively used during research investigations among a specific population (13). The utility of these instruments is vast to identify risk and prompt the need for action. However, a risk score alone (based on a statistically defined threshold) only raises awareness about the existence of a problem. These scales are mostly unidimensional and may not capture the complexity of the situation. Additionally, in most cases, it does not specifically define or describe tailored recommendations for action to increase connectivity among older adults in real time, which raises ethical questions about the benefits of screening/assessment in the absence of action (e.g., additional screening, referral, treatment). While existing tools have merit, they provide a sound and solid foundation for developing and creating new scales, measures, and screeners that are contextually appropriate for use during the COVID-19 pandemic and beyond.

Many clinical and community organizations recognize the importance of addressing social isolation and have started to create or adopt processes to identify risk and attempt to rectify the issue. Despite their respective budding efforts, screening efforts within clinical and community settings remain challenging, as does linking at-risk older adults to needed resources, and services in a timely manner. To date, screening for social isolation risk has not been incorporated into routine clinical care, unlike screening for other key preventable public health risk factors like smoking and high blood pressure (12). Screening for social isolation in clinical care can help improve care, outcomes and population health by providing more precision in diagnoses and treatment, foster better and more shared decision making about treatments that are feasible and appropriate, identify stressful social risk factors so clinicians can connect older adults to helpful community-based public health and social services to address (with their consent), and improve clinical systems' ability to tailor their supports and services to their population's needs (46). At present, clinical-community integration for social isolation screening and referral (i.e., despite the direction, whether the screening originates from the clinical or community sector) is disjointed and at times fragmented. Continuity in screening methods, frequent communication mechanisms, and seamless referral systems are needed to ensure the older adult is identified, monitored, and supported throughout their journey to connectivity. As with other recommended public health screenings (e.g., for depression), it is important that adequate systems for referral, treatment, and follow-up be in place before screening for social isolation risk (47). Without these process, protocols, and mechanisms in place, older adults may be identified for social isolation risk through screening efforts, but the organizations/professionals to which they should be connected, referred, and visited may be unable to sufficiently fill the need. As such, in the time of COVID-19, we are given a unique opportunity to create options, initiate innovations, and improve opportunities to support older persons who are socially disconnected.

## Examples of Feasible and Practical Solutions During COVID-19 and Beyond

In the time of COVID-19 and physical distancing, traditional practices must be rapidly altered and translated to serve and engage older adults, combat social isolation, and facilitate connectivity. Because physical interactions with older adults should be limited, the field must rethink effective solutions for what we will refer here using the term “distanced connectivity.” Distanced connectivity attempts to maintain and repair the fractured or diminished structural, functional, and quality aspects of physical social connectedness through the telephone, computer, or other smart devices. Strategies include the integration of brief and interactive screenings to identify risk and make service referrals during telephonic interactions as well-technology-based intervention delivery and social support. In addition to strategies having the capability to safely reach older adults over time and space, they also have the ability to engage older adults as volunteers and supports during crisis to assist themselves and others to offset social isolation. Within this section, specific examples from the field are provided to illustrate efforts to mitigate the escalating rates of social isolation and associated distress among older adults during the COVID-19 pandemic in the United States.

### Telephonic Reassurance and Engagement

During COVID-19, many organizations are pivoting their efforts to increase distanced connectivity. Some are bolstering existing telephonic efforts while others are altering face-to-face initiatives and services to be delivered telephonically or via the internet. These transitions require the repurposing of personnel and reallocation of funds, which can create strain on the clinical, and community organizations offering the service. To meet the needs of older adults during COVID-19-related physical distancing, many organizations are using telephonic reassurance and engagement efforts. Often this includes having community health workers, social workers, clinicians, and other personnel make telephone calls to older adults for the purposes of checking on their general well-being, identifying needs, engaging them cognitively, offering an opportunity for socializing, and linking them to available services and resources. Telephonic reassurance and engagement efforts can take many forms from brief, unstructured interactions to longer, structured activities with specified objectives. Regardless of the format, effective distanced connectivity via telephone can improve the functional and quality aspects of social connectedness.

An inter-sectorial clinical-community example of a telephonic reassurance and engagement solution during COVID-19 includes a pilot in Maryland. This effort uses care coordinators and volunteers at an Area Agency on Aging (AAA) to call older adults who are members of the senior center or who have been referred by local clinical partners for services. Because face-to-face services are limited, the AAA is making structured calls to identify needs related to nutrition, caregiving, and other social determinants of health. Integrated into a battery of measures and talking points is the *Upstream Social Isolation Risk Screener* (U-SIRS). Completed telephonically in an interview format in Maryland, the U-SIRS is a 13-item brief screener to measure upstream social isolation risk among community-dwelling older adults and link them to appropriate resources, services, and programs. Designed as an interactive and actionable tool, the U-SIRS can be completed independently by an older adult, but its potential impact is heightened when completed with or alongside professionals and community navigators (e.g., clinical organizations and healthcare professionals, community-based organizations, community health workers and promotores, evidence-based program deliverers). The U-SIRS lives on an electronic platform (i.e., can be completed on a computer, tablet, and/or smartphone) to facilitate a tailored screening experience. After completion, the older adult's responses are used to generate a custom report in real-time, which can then be saved or shared with others. Risk level is identified using a stoplight analogy [i.e., high [red], medium [yellow], and low [green] risk]. In the time of COVID-19, the community navigator reviews the list of recommended services and programs, prioritizes them in order of need (and what is available given closures), and assists to make linkages to local services and resources that best match the older adult's needs. Follow-up calls will occur to reassess risk, service utilization, and need. To date, hundreds of older adults have been engaged with the U-SIRS in Maryland (Maintaining Active Citizens—Maryland Living Well-Center of Excellence) and other states during the COVID-19 pandemic.

### Virtual Program and Service Delivery

Given the heightened COVID-19 risks for older adults, face-to-face delivery of evidence-based health and wellness programs has temporarily ceased during the pandemic. These evidence-based programs offer older persons access to quality community-and home-based supports to prevent falls, encourage physical activity, promote mental health, support caregivers, and self-manage multiple chronic conditions. Thus, these behavioral interventions reach older underserved communities where they live, work, pray, and play and provide important supports for persons with limited or no access to health care (48)–the same older communities who are vulnerable for COVID-19. Given the previous widespread availability of these programs nationwide (49, 50), their temporary closure means that thousands of older adults are unable to attend one-on-one and small group workshops to learn about disease self-management, fall prevention, physical activity, and many other topics. Typically delivered in various settings (e.g., healthcare organizations, residential facilities, senior centers, faith-based organizations), this service interruption is unfortunate because these programs provide older adults with the valuable information and support as well as the ability to facilitate social interactions with peers. As such, the Administration on Aging (ACL) and National Council on Aging (NCOA) have responded with recommendations for the aging services workforce to maintain distanced connectivity with older adults (see details at https://acl.gov/COVID-19 and https://www.ncoa.org/covid-19-resources-for-professionals). A coordinated set of resources, toolkits, webinars, factsheets, and other communications have been released to help organizations pivot their efforts to deliver evidence-based programs and services virtually (e.g., asynchronous learning independently, teleconferencing in one-on-one or group formats) or in mailed self-learning format. Efforts to transition face-to-face delivery modalities to virtual and mail-based offerings are an attempt to provide older adults with the services they need and maintain interaction and engagement during physical distancing.

Many examples exist of evidence-based programs that have been translated for virtual delivery (https://www.ncoa.org/news/ncoa-news/center-for-healthy-aging-news/track-health-promotion-program-guidance-during-covid-19). One is the Program to Encourage Active, Rewarding Lives (PEARLS) (51). PEARLS is appropriate for COVID-19 times because it addresses late-life depression symptoms, which are risk factors and consequences of social isolation and loneliness. PEARLS is being evaluated with funding from AARP Foundation as an intervention to improve social connectedness for low-income older persons. PEARLS is traditionally a home-based collaborative care model that trains front-line social service providers to teach problem-solving and activity planning skills help older persons create a “new normal” as they age in order to minimize symptoms of depression and improve social connections through activities and relationships (51). In March 2020, when shelter-at-home orders and other public health guidelines required social service agencies to provide care remotely, PEARLS organizations began offering PEARLS by phone or video-conferencing plus mailed materials (i.e., telePEARLS) based on organizational, provider, and participant accessibility, feasibility, and appropriateness. Older PEARLS participants are benefiting from PEARLS calls to: (1) get emotional, social, and instrumental support; (2) identify new ways of connecting socially in physically distanced times; and (3) learn new skills to reduce anxiety, depression, and stress as well as feelings of social isolation and loneliness. This suggests that virtual delivery of evidence-based programs like PEARLS can reach older marginalized persons to manage chronic physical and mental health conditions, access up-to-date COVID-19 information and essential services, such as food and medications, and feel more connected in times of physical distancing.

## Discussion

Prior to the threat of catching a virus that affects older adults more severely than younger people, many older people were seen as active, continuing to work, care for others, volunteer, and engage with family and friends. Nonetheless, the prevalence of social isolation, loneliness, and depression were becoming increasing acknowledged as hidden problems within the aging population. Social isolation is becoming intensified and complicated during the COVID-19 pandemic. While the newly-required physical isolation provides protection against the virus, social isolation has a range of negative consequences that may be amplified by the stress and uncertainty of the contemporary reality. Existing and emerging efforts to combat social isolation can be strategically modified to combat the COVID-19 Social Connectivity Paradox. In this unprecedented time of physical distancing, providers of all types are recognizing the limits to service accessibility and are creating innovative solutions. Older adults can still remain socially connected despite remaining physically distanced (52). Distanced connectivity that serves older adults most vulnerable to both COVID-19, and the devastating effects of social isolation, must be central to those solutions.

The importance of screening for social isolation and limited connectedness cannot be underscored enough. Screening for risk in clinical and community settings is essential, but screening and assessments become more powerful if they are linked to specific and purposeful action. Most measures are static and were developed in non-COVID times; therefore, efforts are needed to better understanding how to recalibrate the sensitivity of risk identified with these assessments in the context of pandemic precautions and restricted social interaction. For example, as anecdotally documented in the U-SIRS implementation, many older adults who had low social isolation risk prior to COVID-19 are now reporting medium or high social isolation risk because of limited connectivity based on physical distancing and stay-at-home and shelter-in-place orders. Further, rather than using a single measure for social isolation or associated issues of connectivity during COVID-19 (and generally), multiple measures should be employed simultaneously, in concert, to paint a more comprehensive picture of the social isolation and the related needs of the older adult. Additionally, social isolation screening efforts should not only occur once; rather, they should be ongoing and repeated to monitor improvement.

Spurred by the conditions of COVID-19, interventions employed to improve social connectedness should target the underlying mechanisms of change (53). with documented evidence of the ability to reduce isolation and loneliness. Masi et al. (54) offer a user typology for selecting appropriate interventions based on what the intervention is targeting. Effective interventions are those that help with improvement of social skills, enhance social support, increase opportunities for social interactions, and address maladaptive social cognition. Multidimensional screening for low social connectedness can be helpful to identify what aspects of social relationships are missing in the lives of older adults, which can then guide intervention selection appropriate for each older person.

Increasingly, the aging social services network is being recognized for its important role in providing quality, accessible health, and social care to older underserved and/or marginalized persons such as those experiencing poverty, living alone, providing caregiving, and living with physical disabilities (55). During the pandemic, social service organizations are working to provide essential access to older communities in need. Organizations are seeking effective ways to provide support remotely, and older persons are looking for connections to maintain their health, get their basic needs met, and obtain accurate information. Offering evidence-based interventions by telephone or video-chat offers a critical opportunity to learn best practices for offering tele-services that lessen the negative physical, social, and mental impacts of COVID-19 (56). Leading Age's *Social Connectedness and Engagement Technology Tool* provides important guidance for what products are currently available to help organizations choose platforms that fit the needs of their organization and community (57).

The benefits to distanced connectivity via telephonic and virtual service delivery and interactions are undeniable; however, these tele-services are not always accessible to older underserved and/or marginalized communities and social service organizations. Many rapidly emerging strategies to promote distanced connectivity may exacerbate existing digital divides (58, 59). For many older persons, access to reliable internet is limited, and former sources of connectivity such as libraries and senior centers are unavailable. Even if access is available, barriers exist to older adults using technology, including limited technological literacy and negative attitudes about ease of use and security issues (60). COVID-19 may necessitate both the universal access to reliable, broadband internet and ways to improve accessibility, feasibility, and appropriateness of technology for older persons because physically distanced times require virtual ways to connect and access resources.

## Conclusion

The COVID-19 pandemic has highlighted that social isolation is a major public health issue and remaining physically distant can paradoxically be both protective and harmful to older adults. The pandemic also underscores the need for clinical and community-based organizations to unite and form inter-sectorial partnerships to maintain the provision of services and programs for engaging and supporting older adults during this difficult time of physical distancing and shelter-in-place and stay-at-home orders. Older adults can themselves be mobilized and capitalized upon as volunteers and supports so they can remain engaged, combat social isolation risk, and facilitate connectedness among their families and peers. The aging social services network provides a vital infrastructure for reaching older underserved and/or marginalized persons across the U.S. to reduce social isolation. Pre-COVID-19, awareness about the pervasiveness and seriousness of social isolation had begun to spur inter-sectoral partnership and coordinated community action to address its enormous human and financial tolls. These efforts are now more critical than ever because many older adults know they must physically isolate; however, they may not know the importance of maintaining strong social connections or have the tactics or ability to do so virtually. This article provides a perspective about the current situation during the pandemic. Yet, more awareness by the professional and lay communities, as well as more detailed data, are needed to identify the short- and longer-term consequences of COVID-19, as well as the short- and longer-term benefits of distanced connectivity efforts, on social isolation, loneliness, and depression among older adults.

## Author Contributions

MS conceptualized, wrote, and revised the manuscript. LS wrote and revised the manuscript. EC critically revised the manuscript. All authors are accountable for the content of the work and approve the final version of the manuscript.

## Conflict of Interest

The authors declare that the research was conducted in the absence of any commercial or financial relationships that could be construed as a potential conflict of interest.
